# Impact of sub-inhibitory antibiotics on fibronectin-mediated host cell adhesion and invasion by *Staphylococcus aureus*

**DOI:** 10.1186/1471-2180-11-263

**Published:** 2011-12-14

**Authors:** Jean Philippe Rasigade, Abdelmalek Moulay, Yannick Lhoste, Anne Tristan, Michele Bes, François Vandenesch, Jerome Etienne, Gerard Lina, Frederic Laurent, Oana Dumitrescu

**Affiliations:** 1 INSERM, U851, Lyon 69008, France; 2 Centre National de référence des Staphylocoques, Faculté de Médecine Lyon Est, Université de Lyon, Lyon 69008, France; 3 Centre National de Référence des Staphylocoques, 7 rue Guillaume Paradin, 69372 Lyon cedex 08, France

## Abstract

**Background:**

*Staphylococcus aureus *is a well-armed pathogen prevalent in severe infections such as endocarditis and osteomyelitis. Fibronectin-binding proteins A and B, encoded by *fnb*A/B, are major pathogenesis determinants in these infections through their involvement in *S. aureus *adhesion to and invasion of host cells. Sub-minimum inhibitory concentrations (sub-MICs) of antibiotics, frequently occurring *in vivo *because of impaired drug diffusion at the infection site, can alter *S. aureus *phenotype. We therefore investigated their impact on *S. aureus *fibronectin-mediated adhesiveness and invasiveness.

**Methods:**

After *in vitro *challenge of *S. aureus *8325-4 and clinical isolates with sub-MICs of major anti-staphylococcal agents, we explored *fnb*A/B transcription levels, bacterial adhesiveness to immobilised human fibronectin and human osteoblasts in culture, and bacterial invasion of human osteoblasts.

**Results:**

Oxacillin, moxifloxacin and linezolid led to the development of a hyper-adhesive phenotype in the fibronectin adhesion assay that was consistent with an increase in *fnb*A/B transcription. Conversely, rifampin treatment decreased fibronectin binding in all strains tested without affecting *fnb*A/B transcription. Gentamicin and vancomycin had no impact on fibronectin binding or *fnb*A/B transcription levels. Only oxacillin-treated *S. aureus *displayed a significantly increased adhesion to cultured osteoblasts, but its invasiveness did not differ from that of untreated controls.

**Conclusion:**

Our findings demonstrate that several antibiotics at sub-MICs modulate fibronectin binding in *S. aureus *in a drug-specific fashion. However, hyper- and hypo- adhesive phenotypes observed in controlled *in vitro *conditions were not fully confirmed in whole cell infection assays. The relevance of adhesion modulation during *in vivo *infections is thus still uncertain and requires further investigations.

## Background

*Staphylococcus aureus *is a prevalent and dangerous pathogen in humans, causing a wide range of infections. The initial step of suppurative infections, such as infective endocarditis or osteomyelitis, involves bacterial adhesion to the extracellular matrix and cell surface of the host. Several microbial factors involved in this adherence are present in *S. aureus *[1]. Among these factors, collectively designated as microbial surface components recognising adhesive matrix molecules (MSCRAMMs), fibronectin-binding proteins A and B (FnBPA/B) play a major role in *S. aureus *pathogenicity through their ability to bind fibronectin and fibrinogen and to initiate integrin-mediated intracellular uptake of the bacteria by non-professional phagocytes such as endothelial cells or osteoblasts [2,3]. The invasion of host cells by *S. aureus *eventually leads to the formation of an intracytoplasmic reservoir, where bacteria remain protected from the action of cell- and antibody-mediated immune response and from that of most antimicrobial agents. This bacterial sanctuarisation makes successful treatment even more challenging and paves the way for infection relapse [4].

A peculiar difficulty to be faced in treating deep-seated infections is the risk of impaired diffusion of antimicrobial agents at the infection site, where they would only achieve sub-inhibitory concentrations. *S. aureus *strains challenged with such antibiotic concentrations have been shown to exhibit altered phenotypes depending on the molecule tested, including down- or up-regulation of virulence factor expression. For example, beta-lactams enhance the secretion of virulence factors such as the Panton-Valentine leukocidin and alpha haemolysin, while clindamycin or linezolid exert an inhibitory effect [5-8]. However, most studies on the antibiotic-mediated modulation of protein expression by *S. aureus *have focused on secreted exotoxins, and less is known about this modulation with respect to MSCRAMMS, including FnBPA/B.

In the present study, we aimed to investigate the impact of sub-inhibitory concentrations of major anti-staphylococcal agents on the adhesion and invasion phenotypes of *S. aureus*. After *in vitro *challenge of *S. aureus *reference strain 8325-4 and clinical isolates with antibiotics, we explored the following: (i) mRNA expression levels of the *fnb*A and *fnb*B genes, which encode FnBPA and B, respectively; (ii) bacterial adhesiveness to immobilised human fibronectin and human osteoblasts in culture; and (iii) bacterial invasion of human osteoblasts.

## Methods

### Bacterial strains

The bacterial strains used in this study are summarised in Table 1. Laboratory strain 8325-4 and its Δ*fnb*A/B derivative DU5883 were used as a control for *fnb*A/B [9]. Clinical isolates were characterised for the presence of the *fnb*A, *fnb*B, *agr*1-4 and *mec*A genes by PCR as previously described [10,11], and MLST was performed as described by Enright et al. to identify their genetic background [12].

**Table 1:** Characterisation of bacterial strains used in this study

Strain	Type	*fnb*A	*fnb*B	*mec*A	*agr*	ST^a^
8325-4	laboratory strain	+	+	-	1	8

DU 5883	8325-4 Δ*fnb*A/B [9]	-	-	-	1	8

ST2008 1028	clinical isolate, osteomyelitis	+	+	-	3	30

ST2008 0563	clinical isolate, osteomyelitis	+	+	-	1	8

HT2001 0390	clinical isolate, endocarditis	+	+	-	3	1

HT2001 0594	clinical isolate, endocarditis	+	+	-	2	15

^a^Sequence Type (ST) was determined as described by Enright et al. [12]

### Antibiotics and MIC determination

The antibiotics used in this study were as follows: oxacillin, gentamicin, clindamycin, rifampicin and vancomycin purchased from Sigma-Aldrich (L'Isle d'Abeau, France); linezolid provided by Pfizer (Amboise, France); and moxifloxacin provided by Bayer (Wuppertal, Germany). Minimal inhibitory concentrations were determined by broth microdilution assay as recommended by the Clinical Laboratory Standards Institute (CLSI) standards [13].

### Bacterial cultures

The strains were cultured on trypticase blood agar plates and incubated overnight at 37°C. Isolated colonies were resuspended in 5 ml brain heart infusion (BHI) in glass tubes (AES Chemunex France) and adjusted to 0.5 McFarland turbidity, corresponding to 10^8 ^CFU/ml, as confirmed by bacterial count. Bacterial suspensions were cultivated at 37°C with 300 rpm gyratory shaking. After 1 h, antibiotics were added to the culture medium at a concentration of half the MIC, and the incubation was continued for 2 additional hours to reach the mid-exponential phase. McFarland turbidity was measured at the end of the incubation step to determine the impact of antibiotics treatment on bacterial density. Aliquots were then taken, and cellular pellets were prepared as described below for total RNA extraction, the microplate adhesion assay, and the whole cell adhesion and invasion assay.

### Relative quantitative RT-PCR

Aliquots of 1 mL of the *S. aureus *8325-4 cultures were centrifuged at 13,000 g, and the pellets were washed with 1 mL of 10 mM Tris buffer and adjusted to an optical density at 600 nm (OD600) of 1, corresponding to approximately 1 × 10^9 ^*S. aureus *cells/mL. One mL of adjusted and washed bacterial suspension was centrifuged at 13,000 g, and the pellets were treated with lysostaphin (Sigma-Aldrich) at a final concentration of 200 mg/L. The total RNA of the pellets was then purified using the RNeasy Plus Mini Kit (Qiagen) according to the manufacturer's instructions. The RNA yield was assessed by UV absorbance, and 1 microgram of total RNA was reverse transcribed using the Reverse Transcription System (Promega) with random primers, as recommended by the provider. The resulting cDNA was used as the template for real-time amplification of *gyr*B, *fnb*A and *fnb*B using specific primers (Table 2). The relative amounts of the *fnb*A and *fnb*B amplicons were determined by quantitative PCR relative to a *gyr*B internal standard, as described elsewhere [14]. The calibrators in our study were the transcripts from the *S. aureus *8325-4 strain grown without antibiotics, normalised with respect to *gyr*B transcription level. *gyr*B expression was not modified by sub-inhibitory antibiotics, thus allowing its use as an internal control. The relative fold changes in the *fnb*A and *fnb*B expression levels were calculated using the 2^-ΔΔCt ^method using the RealQuant software (Roche Diagnostics).

**Table 2:** Primers used for qRT-PCR in this study

Primer name	5' 3' sequence	Reference
*gyr*B F	GGTGGCGACTTTGATCTAGC	[14]

*gyr*B R	TTATACAACGGTGGCTGTGC	[14]

*fnb*A F	ATTGAGACATTTAATAAAGCGA	[15]

*fnb*A R	TTTTGAATAATCGGACCATT	[15]

*fnb*B F	CACCGAAAACTGTGCAAGCA	[16]

*fnb*B R	TTCCTGTAGTTTCCTTATCAGCAACTT	[16]

### Microplate adhesion assay

Flat-bottom 96-well plates (Immuno Nunc) were coated with 50 μg/mL plasma human fibronectin (Sigma-Aldrich) and incubated overnight at 4°C. Coated plates were inoculated with 200 μL per well of bovine serum albumin BSA and incubated for 20 min at 37°C, and then each well was washed 3 times.

Aliquots of the bacterial cultures described above were centrifuged at 13,000 g for 10 min, and the cellular pellets were washed and resuspended in PBS (Dulbecco's Phosphate Buffered Saline, Sigma-Aldrich). Bacterial suspensions were adjusted to an OD600 of 1, corresponding to approximately 1 × 10^9 ^*S. aureus *cells/mL. One hundred μL of each bacterial suspension was incubated in 3 different wells of the fibronectin-coated plate for 45 min at 37°C with mild shaking. Each well was washed 3 times with PBS to remove non-adherent bacteria. Adherent bacteria were fixed with glutaraldehyde (2.5% v/v in 0.1 mol/L PBS) for 2 h at 4°C and then stained with crystal violet (0.1% m/v) for 30 min at room temperature. Excess stain was rinsed off with Triton X100 solution (0.2% v/v, H_2_O), and the plates were dried at room temperature. Bacterial adhesion to fibronectin was assessed spectrophotometrically (Spectrophotometer MR5000, Dynatec) by determining the optical density at 570 nm (OD570). The results were expressed as the mean ± standard deviation based on triplicates. To assess the potential confounding role of antibiotics-induced reduction of bacterial density in our model, we also searched for a correlation between n-fold changes in bacterial densities and fibronectin binding levels in antibiotics-treated strain 8325-4, as compared to the untreated control.

### Cell culture

All cell culture reagents were purchased from GIBCO (Paisley, UK). The human osteoblastic cell line MG-63 (LGC Standards, Teddington, UK) was grown in Dulbecco's modified Eagle medium (DMEM) containing 2 mM L-glutamine and 25 mM HEPES, 10% foetal bovine serum (FBS) and 100 U/mL penicillin and streptomycin (culture medium) at 37°C and 5% CO_2_. Cells were subcultured twice a week and used up to passage 10 after thawing.

### Adhesion and invasion assay with human osteoblasts

MG-63 cells were seeded at 50,000 cells/well in 24-well plates and incubated at 37°C with 5% CO_2 _for 48 h in culture medium. *S. aureus *strain 8325-4 was treated with sub-inhibitory concentrations of oxacillin, linezolid or rifampicin as described above and then washed and resuspended in antibiotic-free culture medium. The untreated *S. aureus *strain DU5883 (isogenic mutant of strain 8325-4 deleted for the genes *fnb*A/B) was used as a negative control. MG-63 cells were washed twice with DMEM and were infected with bacterial suspensions at a multiplicity of infection of approximately 50:1, as confirmed by bacterial count. Cells were incubated for 2 h at 37°C to allow for adhesion and internalisation of the bacteria and then washed twice with DMEM to remove unbound bacteria. For the adhesion assay, cells were analysed using osmotic shock in pure water and extensively pipetted to achieve full release of cell-associated bacteria. For the invasion assay, infected cells were further incubated for 1 h in culture medium containing 200 mg/L gentamicin to kill extracellular bacteria but not internalised bacteria. Cells were then washed twice in DMEM and analysed as described above to release internalised bacteria. For both the adhesion and invasion assays, viable bacteria in cell lysates were enumerated by plate counting on agar. The number of adherent bacteria was calculated by subtracting the number of internalised bacteria from the number of total cell-associated bacteria. The results were expressed as the mean ± standard deviation of the percentage of recovered internalised or adherent bacteria with respect to inoculated bacteria, derived from four independent experiments performed in duplicate.

### Statistical analysis

The statistical analyses were based on the use of one-way ANOVA followed by the a posteriori Dunnett's test. Correlation analysis was performed using Spearman's rank correlation coefficient. The level of statistical significance was set at 0.05. The tests were carried out with SPSS for Windows version 12.0 software.

## Results

### Susceptibility to antibiotics of bacterial strains cultured in BHI

We first examined the influence of the experimental conditions on the MIC values of tested strains. The oxacillin, gentamicin, vancomycin, clindamycin, linezolid, moxifloxacin and rifampin MICs were determined using CLSI recommendations and compared to those obtained with BHI inoculated with 5 × 10^5 ^CFU/mL (Table 3). MICs in BHI were of the same magnitude as those obtained in Mueller-Hinton, therefore we used BHI medium for the rest of this study.

**Table 3:** MICs of antibiotics tested in BHI medium against 6 selected *S. aureus *strains

	MIC (mg/L) in BHI medium^a^					
	
Antibiotic	8325-4	DU5883	ST2008 1028	ST2008 0563	HT2000 0594	HT2001 0390
Oxacilllin	0.25	0.25	0.25	0.25	0.25	0.25

Gentamicin	1	1	1	1	1	1

Vancomycin	2	2	1	1	2	2

Clindamycin	0.15	> 128^b^	0.30	0.30	0.30	0.30

Linezolid	1	1	1	1	1	1

Rifampicin	0.006	0.006	0.006	0.006	0.006	0.006

Moxifloxacin	0.12	0.12	0.12	0.12	0.12	0.12

^a^MICs were determined with a standard microdilution method recommended by the CSLI in BHI medium inoculated with 5 × 10^5 ^CFU/mL ^b^DU5883 strain is resistant to clindamycin as it harbours the *erm*B gene [9]

### Effect of antibiotics on *S. aureus *adhesion to fibronectin-coated microplates

**Figure 1:**
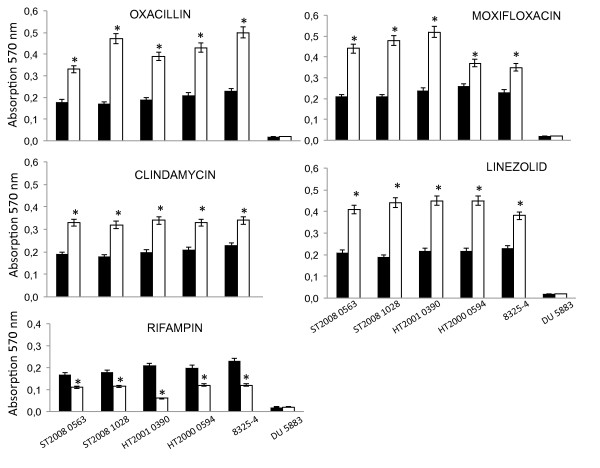
**Effect of antibiotics on the adhesion to human fibronectin**. Exponential growth cultures of *S. aureus *laboratory strains 8325-4 and DU5883 and clinical isolates ST2008 1028, ST2008 0563, HT2000 0594 and HT2001 0390 were treated or not treated with 1/2 of the MIC of antibiotics (oxacillin, moxifloxacin, clindamycin, linezolid or rifampicin) and assayed for adhesion to fibronectin-coated microplates, as described in Methods section. The results are OD570 nm values reflecting bacterial adhesion to fibronectin. The values were obtained from 3 different wells previously incubated with the same bacterial suspension, and adhesion is expressed as the mean ± standard deviation (dark bars for untreated cultures and white bars for antibiotic treated cultures; results from three different experiments). *Asterisk *= significantly different from the control (corresponding isolate grown without antibiotic), with a *P *value of 0.05 by one-way analysis of variance followed by a posteriori Dunnett's test.

We determined the influence of antibiotics on the adhesion of *S. aureus *to fibronectin using a fibronectin-coated microplate adhesion assay. We tested standardised bacterial suspensions of 6 strains previously cultured without antibiotics or with 1/2 MIC oxacillin, gentamicin, vancomycin, clindamycin, linezolid, rifampicin or moxifloxacin. As shown in Figure 1, the adhesion to fibronectin was differentially modulated by the antibiotics. Oxacillin-, moxifloxacin-, clindamycin- and linezolid-treated bacteria displayed increased binding to fibronectin. This effect was observed for all strains tested except *fnb*A/B-negative DU5883. The increase in amplitude of fibronectin binding was strain-dependent. Oxacillin treatment increased fibronectin binding from 1.8- to 2.7-fold relative to the untreated control; moxifloxacin treatment increased binding from 1.4- to 2.3-fold; clindamycin treatment increased binding from 1.5- to 1.8-fold; and linezolid treatment increased binding from 1.6- to 2.3-fold, depending on the tested strain. By contrast, fibronectin binding was significantly reduced after rifampicin treatment. The decrease was strain-dependent and ranged from 1.5- to 3.5-fold compared to the untreated control. Vancomycin and gentamicin had no effect on bacterial adhesion to fibronectin-coated plates (data not shown). Antibiotics-induced reduction in bacterial density had no significant confounding effect on fibronectin binding in our model, as demonstrated by the absence of correlation between n-fold changes in bacterial density and fibronectin binding in antibiotics-treated strain 8325-4 (Additional File 1). The DU5883 strain, defective for *fnb*A and *fnb*B genes [9], did not adhere to fibronectin-coated plates in any condition (with or without antibiotics). Clindamycin could not be tested with the DU5883 strain as it harbours the *erm*B gene and therefore is resistant to clindamycin (Table 3).

### Effect of antibiotics on fnbA and fnbB mRNA levels

**Figure 2:**
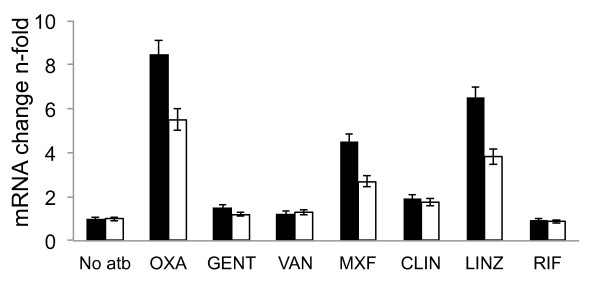
**Effect of antibiotics on *fnb*A and *fnb*B mRNA levels**. Exponentially growing cultures of *S. aureus *8325-4 were treated for 2 h with no antibiotics or with 1/2 the MIC of oxacillin, gentamicin, vancomycin, moxifloxacin, clindamycin, linezolid or rifampicin. Samples of each culture were taken and adjusted to an OD600 of 1 and then used for total RNA extraction and subsequent reverse transcription with random primers, as described above. The cDNA obtained was used as the template for LightCycler PCR with specific *fnb*A, *fnb*B and *gyr*B primers. Relative quantification was performed by reporting it relative to *gyr*B expression, as described elsewhere [14]. The results are expressed as the n-fold variation of *fnb*A (*white bars*) and *fnb*B (*black bars*) mRNA levels in the presence of each antibiotic relative to the growth of no antibiotic control levels. The values are the means ± standard deviations (four different experiments). A change in mRNA level was interpreted as significant if greater than 2-fold variation.

We explored the effect of antibiotics on mRNA expression levels of the *fnb*A and *fnb*B genes which encode FnBPA/B. The *fnb*A and *fnb*B mRNA levels in exponential phase cultures of *S. aureus *8325-4, grown with 1/2 MIC oxacillin, gentamicin, vancomycin, clindamycin, linezolid, rifampicin or moxifloxacin, were compared to those in an untreated culture and expressed as n-fold variation. A change in mRNA level was interpreted as significant if there was greater than 2-fold variation. As shown in Figure 2, oxacillin induced a 5.5-fold increase in the *fnb*A mRNA level and 8.5-fold increase in the *fnb*B mRNA level; moxifloxacin induced a 2.7-fold increase in the *fnb*A mRNA level and 4.5-fold increase in the *fnb*B mRNA level; and linezolid induced a 3.8-fold increase in the *fnb*A mRNA level and 6.5-fold increase in the *fnb*B mRNA level. No significant changes in fibronectin binding gene expression were observed for gentamicin, vancomycin, clindamycin or rifampicin.

### Effect of antibiotics on the adhesion and invasion of osteoblastic cells

**Figure 3:**
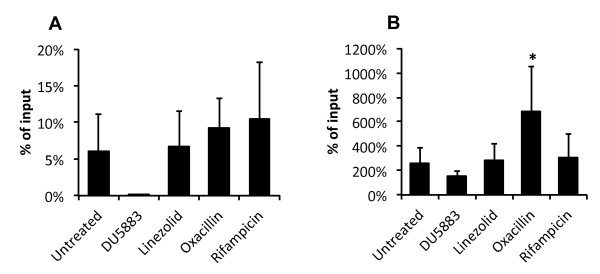
**Effect of antibiotics on *S. aureus *adhesion to and invasion of human osteoblasts**. MG-63 osteoblastic cells were infected for 2 h at approximately 50 bacteria/cell with *S. aureus *strain 8325-4, pre-treated or not (untreated control) with 1/2 MIC linezolid, oxacillin or rifampicin, and *S. aureus *strain DU5883 lacking *fnb*A and *fnb*B (negative control). To enumerate cell-associated bacteria, infected cells were washed twice to discard unbound bacteria and analysed by osmotic shock in pure water, and then, suitable dilutions of the lysates were plated on agar. The same procedure was used to quantify intracellular bacteria, except that the cells were incubated for 1 h with 200 mg/L gentamicin before the lysis step to kill extracellular bacteria. Adherent bacteria were calculated by subtracting intracellular bacteria from cell-associated bacteria. The results were expressed as the means +/- standard deviation of the percentage of recovered internalised (**a**) or adherent (**b**) bacteria with respect to inoculated bacteria derived from four independent experiments performed in duplicate. *Asterisk *= significantly different from the control (corresponding isolate grown without antibiotic), with a *P *value of 0.05 by one-way analysis of variance followed by a posteriori Dunnett's test.

We investigated whether antibiotic-mediated modulation of the expression of *fnb*A and *fnb*B induced changes in *S. aureus *adhesion to and invasion of host cells in an *ex vivo *model. We infected osteoblastic MG-63 cells with the following: (i) *S. aureus *8325-4, either untreated or treated with 1/2 MIC linezolid, oxacillin or rifampicin and (ii) invasion-deficient strain DU5883. We then compared the amounts of adherent and internalised bacteria recovered after 2 h. As shown in Figure 3, oxacillin-treated *S. aureus *exhibited significantly increased adhesion (682 ± 374%) compared to untreated *S. aureus *(256 ± 128%), whereas the adhesion of bacteria treated with linezolid or rifampicin (279 ± 141% and 306 ± 190%, respectively) did not differ significantly from the untreated control. Strain DU5883 showed a tendency towards impaired adhesion (151 ± 40%) compared to its parental strain 8325-4. With respect to bacterial invasion, bacteria treated with linezolid, oxacillin or rifampicin (6.7 ± 4.9%, 9.2 ± 4.1% and 10.4 ± 7.8%, respectively) did not exhibit significant differences compared to the untreated control (6.0 ± 5.1%), while host cell invasion was abolished in strain DU5883 lacking *fnb*A and *fnb*B (0.0 ± 0.0%).

## Discussion

Several major findings emerge from this investigation of the impact of sub-inhibitory concentrations of anti-staphylococcal drugs on *S. aureus *adhesion and invasion phenotypes. *S. aureus *binding to human fibronectin and the transcriptional levels of the *fnb*A/B genes encoding the fibronectin-binding proteins were differentially modulated by antimicrobial agents. Oxacillin, moxifloxacin and linezolid treatment led to the development of a hyper-adhesive phenotype, along with an increase in *fnb*A/B mRNA levels relative to the *gyr*B internal standard. The same hyper-adhesive phenotype was induced by clindamycin treatment, although no significant change in *fnb*A/B mRNA levels was observed. Rifampin was the only antimicrobial agent among those tested that significantly inhibited *S. aureus *binding to fibronectin without affecting relative *fnb*A/B transcription profiles. Vancomycin and gentamicin induced no change in either the adhesion phenotype or the *fnb*A/B transcription. *S. aureus *adhesion to and invasion of live eukaryotic cells was also assessed after oxacillin, linezolid or rifampin treatment in an *ex vivo *infection model of cultured human osteoblasts. Oxacillin treatment significantly increased *S. aureus *adhesion but not invasion, while no significant change in adhesion or invasion levels was observed after linezolid or rifampin treatment.

Several recent studies have focused on the influences of sub-inhibitory concentrations of antimicrobial agents on the expression of various virulence factors produced by *S. aureus *and on the various regulation mechanisms involved in this modulation [6,8,17]. Similarly, the expression and surface display of FnBPs are regulated by a complex network of global regulators and stress response pathways that can be triggered by antimicrobial agents in a drug-specific fashion. Fluoroquinolones, by activating the SOS system (a global response system to DNA damage), have been shown to induce *fnb*B up-regulation and fibronectin binding in *S. aureus *through a LexA-RecA-dependant pathway [18]. Moreover, in a rabbit *S. aureus *infection model, moxifloxacin treatment inhibited the expression of *agr *global regulator [19], which acts as a repressor of surface protein expression, including *fnb*A/B, and as an activator of exotoxin expression [20]. Beta-lactams, besides inducing the SOS response system [21], have also been reported to up-regulate virulence factor expression, including *fnb*B, through the two-component system SaeRS [22].

Clindamycin and linezolid are protein synthesis inhibitory agents known to repress exotoxin secretion by *S. aureus *[6-8]. Thus, their positive effect on fibronectin binding in *S. aureus *makes it tempting to speculate that their impact on protein expression involves selective inhibition of *agr*. We recently showed that sub-inhibitory concentrations of linezolid repress early *agr *expression in *S. aureus *[23]. Furthermore, sub-inhibitory concentrations of clindamycin have been shown to decrease *sae*RS expression [24], thus possibly interfering with *fnb*B expression. An alternative explanation for the effects of clindamycin has been reported by Blickwede et al., who observed that *fnb*B mRNA levels were selectively increased after clindamycin treatment and that this increase was due to mRNA stabilisation in the presence of clindamycin [25]. Whether linezolid also affects fnbA/B mRNA levels through mRNA stabilisation remains unknown, and this question merits further investigations.

With respect to sub-inhibitory rifampin treatment, the decrease in fibronectin binding observed here was not accompanied by a transcriptional decrease of *fnb*A/B relative to the internal control *gyr*B, suggesting that fibronectin binding inhibition takes place at the post-transcriptional level. Mechanisms underlying the effects of rifampin in this context are still to be elucidated. We speculate that these mechanisms could involve either a decrease of sortase activity, which is responsible for cell wall anchorage of several MSCRAMMs including FnBPs [26,27], or an increase of protease activity, which has been shown to dramatically influence fibronectin-binding in *S. aureus *[28].

Interestingly, fibronectin-binding modulation by oxacillin, linezolid or rifampin only partially correlated with host cell adhesion and invasion under our experimental conditions. Although oxacillin-treated *S. aureus *displayed significantly increased binding to cultured osteoblasts, its invasiveness did not differ significantly from that of the untreated control. Beta-lactams interfere with cell division and induce dramatic changes in staphylococcal morphology even at sub-inhibitory concentrations [29]. Of note, the inhibition of cell separation ultimately leads to the formation of so-called pseudomulticellular staphylococci [30]. These aberrant forms were present following oxacillin treatment under our experimental conditions, whereas bacterial size and morphology were unchanged in bacteria either untreated or treated with rifampin or linezolid, as objectivated by microscopic examination after fluorescence staining of the cell wall (data not shown). It is likely that the larger size of pseudomulticellular staphylococci hampers their internalization by osteoblasts, which could negatively compensate the increase in adhesiveness induced by oxacillin. In the same way, we failed to identify a change in adhesion and invasion phenotypes after linezolid or rifampin treatment. A putative explanation for these discrepancies between phenotypes observed under both controlled *in vitro *conditions and more complex *ex vivo *infection assays is adhesin redundancy. Although FnBPs play a major role in *S. aureus*-host cell interactions, whole cell adhesion involves several other MSCRAMMs [31], which are also likely regulated by antibiotics and thus could hamper or cancel the effects of FnBPs modulation. This outcome is illustrated by our finding that strain DU5883 lacking *fnb*A/B still adhered significantly to cultured osteoblasts. The same is probably true with respect to *S. aureus *invasiveness, although a more limited number of factors are involved along with FnBPs in the cell invasion process. FnBPs are required and sufficient for host cell invasion [27], as confirmed in our model by the observation that invasiveness was abolished in strain DU5883. However, the multifunctional protein *eap*, which also binds fibronectin, acts additively with FnBPs to mediate host cell invasion in *eap*-positive strains such as 8325-4 [32] and can partially compensate for loss of FnBP functions [27]. Additional studies are warranted to determine whether compensatory mechanisms occur to sustain host cell invasion, despite rifampin-mediated FnBP expression decrease.

## Conclusions

It has long been well-established that the choice of antimicrobial agents in therapy should not solely rely on their respective bactericidal or bacteriostatic activity and pharmacokinetics but should also take into account their influence on bacterial virulence [33,34], including adhesion phenotype. Our results confirm that several anti-staphylococcal agents induce a hyper-adhesive phenotype in *S. aureus *through FnBP up-regulation *in vitro*, while only rifampin inhibits fibronectin binding. However, drug-dependent modulation of adhesion, although unambiguous at the molecular and specific ligand-binding level, was not always significant in our *ex vivo *model. This paradoxical observation is reminiscent of that recently reported by Ythier et al., who demonstrated that *in vitro *adherence to fibronectin of clinical *S. aureus *isolates did not correlate with infectivity in a rat model of endocarditis [35]. While antibiotic-mediated modulation of secreted exotoxins has proved fully relevant in the clinical field with respect to several toxin-associated diseases [33,34], the relevance of adhesion modulation during *in vivo *infections is still uncertain and requires further investigation.

## Authors' contributions

JPR, YL carried out the *ex vivo *adhesion and invasion assays. AM, OD carried out the adhesion and RT-PCR assays. JPR and OD drafted the manuscript. GL, AT, MB participated in the design of the study and performed the statistical analysis. GL, FL, FV, JE conceived of the study, and participated in its design and coordination and helped to draft the manuscript. All authors read and approved the final manuscript.

## Supplementary Material

Additional file 1 **Impact of antibiotics on the growth kinetics of S. aureus strain 8325-4 and correlation analysis between n-fold changes in bacterial density and fibronectin binding**. Panel A. Bacterial suspensions were cultivated with or without antibiotics at half-MIC for 2 h as described above. Growth curves with and without antibiotics are represented as Δ log variations of the bacterial density. Panel B. Antibiotics-treated suspensions of S. aureus 8325-4 were assayed for fibronectin binding as described above. Spearman's rank correlation coefficient was calculated and no correlation was found between the bacterial density changes and fibronectin binding measures. Click here for file
