# Supplementary material for: Impact of sub-inhibitory antibiotics on fibronectin-mediated host cell adhesion and invasion by Staphylococcus aureus
Source: BMC Microbiol. 2011 Dec 14;11:263. doi: 10.1186/1471-2180-11-263 (PMC3264541; doi:10.1186/1471-2180-11-263)
Supplement: Additional file 1 — Impact of antibiotics on the growth kinetics of S. aureus strain 8325-4 and correlation analysis between n-fold changes in bacterial density and fibronectin binding. Panel A. Bacterial suspensions were cultivated with or without antibiotics at half-MIC for 2 h as described above. Growth curves with and without antibiotics are represented as Δ log variations of the bacterial density. Panel B. Antibiotics-treated suspensions of S. aureus 8325-4 were assayed for fibronectin binding as described above. Spearman's rank correlation coefficient was calculated and no correlation was found between the bacterial density changes and fibronectin binding measures. [file 1471-2180-11-263-S1.PDF]

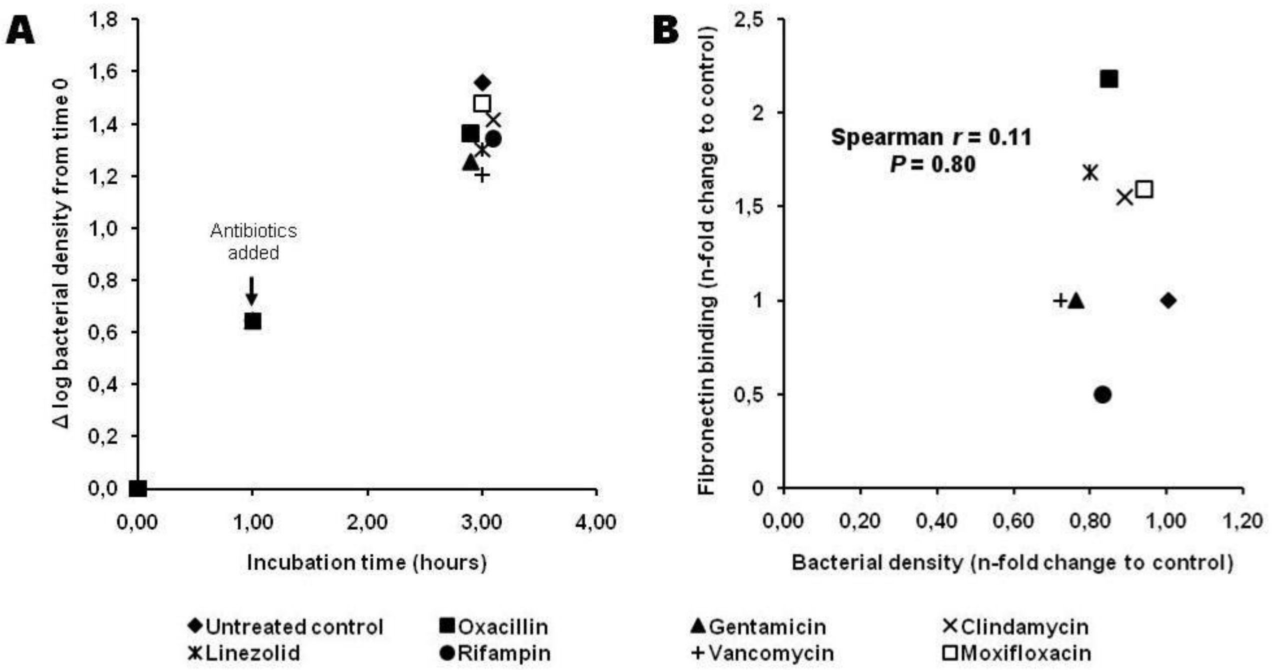

Additional File 1. Impact of antibiotics on the growth kinetics of *S. aureus* strain 8325-4 and correlation analysis between n-fold changes in bacterial density and fibronectin binding. Panel A. Bacterial suspensions were cultivated for 1 h then treated with half-MIC antibiotics for 2 h. Bacterial density was measured at 0, 1 and 3 h using McFarland turbidity scale. Results were expressed as Δ log variations to bacterial density measured at time 0. **Panel B.** Antibiotics-treated suspensions of *S. aureus* 8325-4 were tested for fibronectin binding using a fibronectin-coated microplate assay and spectrophotometric quantification as described in Methods. Spearman's rank correlation coefficient was calculated for n-fold changes in bacterial density and fibronectin binding as compared to the untreated control. No correlation was found between these two variables, thus indicating that antibiotics-induced reduction in bacterial density had no significant confounding effect in our model.
